# Drivers behind the public perception of artificial intelligence: insights from major Australian cities

**DOI:** 10.1007/s00146-022-01566-0

**Published:** 2022-10-03

**Authors:** Tan Yigitcanlar, Kenan Degirmenci, Tommi Inkinen

**Affiliations:** 1 City 4.0 Lab, School of Architecture and Built Environment, Queensland University of Technology, 2 George Street, Brisbane, QLD 4000 Australia; 2 School of Information Systems, Queensland University of Technology, 2 George Street, Brisbane, QLD 4000 Australia; 3 Department of Geography and Geology, University of Turku, Turun Yliopisto, 20014 Turku, Finland

**Keywords:** Artificial intelligence (AI), Public perception, Public policy, Sydney, Melbourne, Brisbane

## Abstract

Artificial intelligence (AI) is not only disrupting industries and businesses, particularly the ones have fallen behind the adoption, but also significantly impacting public life as well. This calls for government authorities pay attention to public opinions and sentiments towards AI. Nonetheless, there is limited knowledge on what the drivers behind the public perception of AI are. Bridging this gap is the rationale of this paper. As the methodological approach, the study conducts an online public perception survey with the residents of Sydney, Melbourne, and Brisbane, and explores the collected survey data through statistical analysis. The analysis reveals that: (a) the public is concerned of AI invading their privacy, but not much concerned of AI becoming more intelligent than humans; (b) the public trusts AI in their lifestyle, but the trust is lower for companies and government deploying AI; (c) the public appreciates the benefits of AI in urban services and disaster management; (d) depending on the local context, public perceptions vary; and (e) the drivers behind the public perception include gender, age, AI knowledge, and AI experience. The findings inform authorities in developing policies to minimise public concerns and maximise AI awareness.

## Introduction

Artificial intelligence (AI) is one of the hot, perhaps the most popular, topics in contemporary social scientific technology research (Yigitcanlar and Cugurullo 2020). As Paschen et al. (2020) described AI broadly is a set of digitalised (computational) agents those can observe and combine information and are also able to act upon that information successfully. In practice, AI is a collective term that includes data capture, analysis, and sharing technologies such as Internet of things (IoT), neural networks, machine learning, and robotics (Zhang et al. 2019; Yigitcanlar et al. 2020a; Alter 2021; Barns 2021).

The first steps in computer system integration and industrial robotics emerged in the 1960s. Their goal was to substitute human labour in the production lines (essentially in automobile industry). Since then, industrial robotics and assembly lines are producing numerous high-end consumer products ranging from electric vehicles to smart home appliances and other applications. Today, also governmental agencies and offices are boosting their operations with AI applications (Kaplan and Haenlein 2020). However, there is an undeniable knowledge gap between public perceptions of AI and what implications does it have on public life (Cui and Wu 2021; Selwyn and Gallo Cordoba 2021).

An easy argument in the current AI research (and utilisation) is highly limited public attitude (perceptions) and the limited role of citizens in technology studies in general (Gao et al. 2020; Regona et al. 2022). Nonetheless, the research is limited as can be verified by looking at ScienceDirect and Scopus databases. A simple Scopus search provided only 126 (as of 29 September 2021) papers with the joint keywords “AI and citizens” in the field of social sciences. The use of alternative phrases such as “AI and perceptions” or “AI and public opinion” provided even lesser number of matches. The obtained topics verify that the majority of the current social scientific AI research concerns applications associated with public administration or private business development and customer intelligence. The field is also new. For instance, 87% (*n* = 110) of the available papers (in the social science category) were published after 2015. Time bias is probably due to the extensiveness and coverage of Scopus that limits the availability of older research papers, but it also verifies the topicality of the AI as a raising trend in social scientific research.

One of the challenges for the public to form a clear opinion on AI technologies is that these technologies are an invisible element of daily life mostly driven by proprietary algorithms. AI technologies are difficult for most people to understand and hence hard for the public to form a clear and accurate opinion on their opportunity and constraints. There is also a major problem in AI research that fails to enlighten the public. To elaborate further, Johnson and Verdicchio (2017, p. 575) see the route of the problem of this limited public understanding of AI as “a confusion about the notion of ‘autonomy’ that induces people to attribute to machines something comparable to human autonomy, and a ‘sociotechnical blindness’ that hides the essential role played by humans at every stage of the design and deployment of an AI system”. According to Crawford and Calo (2016), not engaging the public in the AI research is a blind spot. Hence, AI companies and researchers need to work with the public to advance public understanding and develop some shared standards.

There is strong evidence that AI is becoming rapidly and fundamentally embedded into the modern life. Today, AI applications are being used almost in all sectors in increasing levels. Some examples include, but are not limited to retail, manufacturing, construction, transport, marketing, banking, finance, medicine, mining, engineering, energy sectors, and government services. The main factor that motivates these sectors in AI adoption at large is creating efficiencies through reducing human error, taking physical risks from humans, offering 24 × 7 availability, helping in repetitive jobs, providing digital assistance, making faster decisions, and so on Kabalisa and Altmann, (2021).

Common AI applications, however, reinforce the pervasiveness of the technology, but they are invisible to the public. For instance, there are numerous digital (including traditional and social media platforms) services and applications such as Amazon, Netflix, Facebook, Instagram, and Twitter that plan and execute marketing actions based on their client behaviour big data—where in most cases, the users are not aware of AI algorithms constantly monitoring their behaviours.

The current analytical algorithms are self-learning, thus exerting fundamental principles of AI (Yigitcanlar et al. 2020b). Even they may be far away from science fiction depicted human-like forms of intelligence, and they possess several characteristics that are common in work aiming to iterate essential out of the bulk (e.g. in data processing). Customer (as service user or citizen) profiling tends to direct behaviour towards the desired outcome motivated by the algorithm design.

On the one hand, the current condition of citizen (or end-user) perception and knowledge regarding elements of AI is topical. These issues include for example general awareness of the condition of on data privacy, data management protocols and security, IoT integrations, and interchangeable data sharing (Sutherland 2008). On the other hand, a challenge for the analysis of citizen perceptions is familiarity with the AI technology and its implications.

Survey methods have a long and extensive research tradition in social sciences (Evan and Miller 1969). The golden years for survey research were on the latter part of the twentieth century, and today, conduction of rigid and extensive surveys is both expensive and difficult. There are several reasons including increased sense of privacy, data management requirements, address acquisition, and respondents’ lack of time to answer extensive questionnaires. Keeping this in mind, the paper is based on rigidly collected (extensive) survey data asking questions (perceptions) on highly up-to-date AI. Our approach provides novelty that is reliable, repeatable, and rigid.

The structure of the paper is as follows. Following introduction section, we move to wrap up essential literature analysing the topic of citizen perceptions on technology, including AI. The literature review stresses the importance of diverse AI understanding as it cannot be properly discussed without data analytics, data warehousing, IoT, and other fundamental technologies enabling information economy and society. After the literature background, we move to empirics and present new evidence from Australia. The paper concludes with discussion on the findings and future research directions.

## Literature background

Earlier research has identified the need of multidisciplinary collaborations to assess the role and importance (impacts) of AI in society (e.g. Boyd and Wilson 2017; Theodorou and Dignum 2020; Dahlin 2021; De Neufville and Baum 2021). The basic argument follows the line of thought that AI concerns mainly technological development driven by engineering domains of science, whereas social sciences are focusing on social impacts and uneven developments caused by technological progression. These include, for example to name a few, issues of human rights (Chatterjee and Sreenivasulu, 2022), public health (Masys et al. 2021), general issues of citizen well-being and the future of technologies (Cortes et al. 2021), responsible and sustainable future (Yigitcanlar et al. 2021a), and ethics (Russel et al. 2015). These topics are in the heart of the survey prepared for the study at hand, and we detail these questions in Appendix A (Parts B, C, D, F).

Social scientific research has established that technological progression is, inherently, linked to social condition and acceptance. One of the main societal impacts from AI derives from continuously improving data processing capability or efficiency (Kitchin 2014). AI contributes directly to these evolving decision support systems targeted to improve efficiency and improving worst-case outcomes (e.g. the case of human–machine decision problematic, see Yigitcanlar et al. 2020c; March 2021).

Open and closed innovation mindsets are identifiable in AI systems, as grassroot level (bottom-up, diversified) development tends to favour the former and business-driven (top-down, kernel/silo) the latter (Lewowski and Madeyski 2022; Stahl 2022). From the end-user (citizen) perspective, the provision form is, perhaps, not the most interesting one, but it entails the traditional logics of dichotomies. Thus, AI presence can be achieved with open or closed systems that are produced either publicly or privately, and they can be non-profit or profit-seeking ventures.

AI algorithms evolve constantly. Their main use concerning citizens (or customers) is in data integration and association. This raises questions of data integration (i.e. database combining). In the case of the public sector (Wirtz et al. 2018; Reis et al. 2019), combination of different administrative databases enables highly accurate estimation platform for behaviour probabilities. In several countries, registry and data legislation forbids automated public registry data integration, such as cross-referencing health records, income/taxation records, social fare records, or criminal, legal, and court records, just to name a few examples. These contribute to the trust and privacy issues towards governance and public sector in general.

Machine learning and autonomous decision-making are fundamental goals of AI. The golden aim is justified (data based) decision providing the best solution for each problem. AI should provide efficiency, thus providing better service with lesser cost for both, to the customer and to the service provider. As such, AI-based public service development requires collaboration. This entails critical implications of provision philosophy as numerous public sector services are “in-house” productions or are ad hoc tailored to meet the specific requirements of that public domain. Generally, public services are considered such that they should be independent from a single service provider (i.e. private company with an exclusive contract) that might create liability risks in disagreements (Agarwal 2018). This topical issue concerns risk, trust, and reliability of AI (Appendix A, Parts E, G).

AI implementations have numerous open questions and drawbacks. Cortes et al. (2021) point out a concern regarding “low-quality” data that could have negative impacts on governance quality. Data quality issues and AI algorithms capability to cope with bad quality data will be one of the most important steps in creating an entity (technological) capable of decision-making that has impacts on citizens. Another significant issue concerning city governments (or other regional or territorial legal entities) is that whether they implement the same data management structures horizontally (convergence) or use whatever platform they see fit for their needs (divergence).

The relationship between technology and society dates to the beginning of human civilisation (Yigitcanlar 2016). The survival of humankind depended and still depends on technology, whether it was mechanic in nature before the digital revolution, and digital since then (Allam 2019). While the hazards of misuse of technology, such as nuclear and AI technology in warfare or big brother AI practice of autocratic governments, are acknowledged, Latour (1990) referred to technology as a vehicle that “makes society durable”. This is true in many ways such as green/renewable technologies as a contributor to the sustainable development and climate action efforts (Mendes 2022). Along with this technology, adoption of the public and societal change has been an important research area during the last several decades (Koul and Eydgahi 2018).

Today’s societies produce enormous amounts of data (Ghani et al. 2019). The ever-increasing data enables AI to hold a promise of better services and new business opportunities. It has a significant role for both private and public sectors as they both create new services to meet the evolving demands of the digitalised society (Appendix A, Part H) (Makridakis 2017; Wang et al. 2021). Considering daily living and survey respondents most likely contacts with AI comes from urban technologies (Batty 2018; Cugurullo 2020; Yigitcanlar et al. 2021b). Perhaps the most convenient field includes traffic management and modelling. There are impeccable motivators for AI use—traffic volumes and flows are easy to model due to real-time traffic data. These are practical examples of making everyday life easier (e.g. less time-consuming urban transport), thus creating a positive demand of AI services (Yigitcanlar et al., 2022a). We assume that this has an impact on the survey perception descriptive statistics as well as analytic models.

In the case of emergencies and disasters, AI supported control systems providing more flexibility and modular response systems such as to cope with congestion or accident emergences. Real-time (agile) services are not the only potential application fields for AI (Abduljabbar et al. 2019). As an example, police, fire departments, and search and rescue units are traditional public (emergency) services. AI applications in these departments require highly developed legislation regarding the responsibilities between (possibly numerous) system providers, system management, and personal responsibilities. Coping with emergencies and disasters entail an essential aspect for the future AI (Appendix A, Part I).

Based on the above considerations, we formulate our empirical task to concern complex issue of social construction of AI. Similarly, Inkinen et al. (2018) conducted a reference study on e-service adoption that highlighted the importance of education and age as the main explanative variables determining attitudes and beliefs in e-service use and adaption. Our empirics apply them as well (Appendix A, Part A). In content questions, we emphasise attitudes and views of services as well as public view of trust and privacy towards AI. The data will provide a timely awareness check on the concerns and benefits perceived by the Australian respondents in the largest cities. They include private and public services and their acceptance drivers.

## Methodology

We conducted an online survey to collect data from the public to capture their perceptions on AI. The target areas are selected as the largest capital cities of Australia, namely Sydney, Melbourne, and Brisbane. Individuals living within the areas up to 50 km crow fly distance from CBD of these three cities are targeted to capture responses. The only criterion to qualify for participation to the survey was being 18 years old or over. A professional survey panel recruiting company was hired to recruit participants. The mentioned survey panel company sent the online survey link to 2193 individuals via email at the beginning of May 2020, and 605 valid responses were received within the same month. This figure of 605 is well over the minimum sample size requirement of 385. The minimum sample size is calculated by using the Australian Bureau of Statistics sample size calculator—with 95% confidence level, 0.05 confidence interval, and for the population size of 5 million. This tool is accessed online at https://www.abs.gov.au/websitedbs/d3310114.nsf/home/sample+size+calculator.

Participants were provided with a definition of AI to avoid bias due to misinterpretation. A total of 605 valid responses received from the survey participants, resulting in a response rate of 27.6%. Most of the participants were female (50.2%), 35–44 years of age (20.8%), had a bachelor’s degree (31.9%), were employed (61.5%) mostly in the retail trade industry (9.1%), and were professionals (17.7%), with a gross weekly income of $1,000–1,999 (29.3%). We have also provided salient demographic characteristics of the three study areas to present the survey participants representativeness with the case study areas (Appendix B).

Despite our best efforts to have an ideal representation of the population characteristics of the three case cities in the survey, there occurred some representation differences between the study participant characteristics and the actual resident characteristics of the three case cities—e.g. in our survey 16.5% of the participants were 65 years old and over, where this figure for Australia is 22.9%. This mismatch issue is not an uncommon challenge of survey studies, and thus, along with the others, this limitation is mentioned at the conclusion section of the paper. Additionally, we note that Covid-19 may have an influence on the perceptions of the surveyed populations—circumstances may have made them interact more with technology—as the pandemic and lockdowns have triggered the pace of the digital transformation in our societies (Nagel 2020).

To measure public perceptions of AI about perceived benefits, risks, and trust, we developed constructs by following the lead of the academic literature on AI, public perceptions, and government decision automation (e.g. Fraszczyk and Mulley 2017; Araujo et al. 2020; Peng 2020; Stai et al. 2020; Cui and Wu 2021; Dennis et al. 2021; Kassens-Noor et al. 2021; Selwyn and Gallo Cordoba 2021). All items were measured on a 7-point Likert scale ranging from “disagree” to “agree” for perceived benefits and risks, and “distrust” to “trust” for the trust construct.

To categorise the identified attributes under fundamental dimensions and facilitate the interpretation of the data, we conducted a factor analysis using SPSS. Complementary to the factor analysis, we also conducted a descriptive analysis, where we calculated the level of agreement on investigated attributes. To calculate the level of agreement, “agree” categories were combined to calculate the percentage of agreement, and “disagree” categories were combined to calculate the percentage of disagreement. The percentage of the mid-point was used to report “neutral”. We ranked the investigated attributes on the percentage of agreement and disagreement.

## Results

We conducted the factor analysis to reveal latent variables that cause the covariation between observed variables. To achieve a clean factor structure, we suppressed coefficients under 0.5. Table 1 shows that items loaded higher on their respective factors and cross-loading was not an issue. We calculated Cronbach’s alpha, which confirmed high reliability above 0.9 for each factor. Values above 0.7 indicate strong reliability. Further, we conducted a Kaiser–Meyer–Olkin (KMO) test, which revealed a value of 0.926. The KMO test explains whether all the included variables are adequate for the factor analysis. Values above 0.6 are considered to be acceptable.

**Table 1:** Results of the descriptive and factor analyses (combined dataset for Sydney, Melbourne, and Brisbane)

Attributes	Descriptive analysis					Factor analysis			
	DA%	Neutral%	A%	R-DA	R-A	AI risks	AI trust	AI benefits for urban services	AI benefits for disaster management
AI machines being used to monitor your activity without your permission/knowledge	8.9	22.6	68.4		1	0.812			
AI machines being used to invade your privacy	11.6	22.3	66.1		2	0.804			
AI machines making misdiagnosing illness	13.6	27.6	58.8		5	0.791			
AI machines being unreliable	17.2	30.7	52.1		9	0.785			
AI machines being used for terrorist activity	14.0	26.3	59.7		4	0.784			
AI machines making bias decisions	13.7	29.4	56.9		7	0.774			
AI machines being hacked and stealing/losing large amounts of your private data	11.9	22.3	65.8		3	0.774			
AI machines creating mass unemployment	17.5	25.3	57.2		6	0.753			
AI machines replacing your job	21.8	25.1	53.1		8	0.707			
AI machines turning against and trying to destroy humanity	32.9	27.4	39.7		11	0.701			
AI machines becoming more intelligent than humans	26.3	28.1	45.6		10	0.595			
How do you feel about the corporations using AI?	27.3	36.2	36.5		4		0.817		
How do you feel about the companies developing and commercialising AI?	24.8	39.2	36.0		5		0.814		
How do you feel about the government agencies using AI?	31.6	32.6	35.9		6		0.806		
How do you feel about the use of AI in public spaces?	26.3	35.5	38.2		3		0.796		
How do you feel about the use of AI in your workplace?	25.5	34.5	40.0		1		0.791		
How do you feel about the use of AI in your home?	28.9	32.7	38.3		2		0.777		
AI can help with efficient delivery of urban services	9.9	31.9	58.2		2			0.858	
AI can help local governments monitor and respond to problems associated with urban infrastructure	9.6	29.9	60.5		1			0.849	
AI can free up resources so that local governments can spend more time focusing on resident needs and concerns	11.9	34.7	53.4		6			0.848	
AI can be used to reduce public sector costs with savings to improve other urban services	12.4	33.9	53.7		5			0.840	
AI can be used to reduce public sector costs with savings used to reduce rates and other taxes	13.2	34.0	52.7		7			0.831	
AI can help local governments monitor and respond to the environmental and climate crises	11.1	30.7	58.2		2			0.819	
AI can help improve objectivity in the delivery of urban services	11.2	36.0	52.7		7			0.792	
AI can be used to monitor urban areas and ensure safety and security of all residents	12.4	30.1	57.5		4			0.774	
AI can help in increasing effectiveness and efficiency of planning and preparedness for disasters	25.6	24.3	50.1		3				0.815
AI can help in disaster response and emergency services in rescue operations	26.1	24.3	49.6		5				0.814
AI can help in emergency services in disaster-related information gathering	25.0	21.3	53.7		1				0.805
AI can help in responding queries of the public regarding to disasters	26.8	27.3	46.0		7				0.793
AI can help in determining disaster hotspots and severity	26.6	24.1	49.3		6				0.791
AI can help in predicting disasters, and providing early warnings	26.4	23.1	50.4		2				0.780
AI can help in determining disaster damages and risky constructions and locations	25.5	24.6	49.9		4				0.768
AI can help in social media analytics to obtain public perception on disasters	25.5	31.6	43.0		9				0.763
AI can help in identifying fake news about disasters	26.1	28.8	45.1		8				0.730
AI can be used in gaming applications to increase community disaster awareness	29.9	31.2	38.8		10				0.617
Sum of squared loadings (rotated)						6.419	4.515	6.449	5.957
% of variance explained						18.339	12.899	18.425	17.020
Reliability (Standard Cronbach’s alpha)						0.923	0.951	0.953	0.923
Kaiser–Meyer–Olkin (KMO)									0.926
*N*									605
Extraction method						Principal component analysis			
Rotation method						Varimax with Kaiser Normalisation			

*DA* disagree, *A* agree, *R* rank

A descriptive analysis disclosed that most of the respondents were concerned about risks caused by AI technology. The respondents are mainly concerned about “AI machines being used to monitor your activity without your permission or knowledge” (ranked 1, agree), and about “AI machines being used to invade your privacy” (ranked 2, agree). This issue underlines the increasing concerns of consumers and related permission requests to access personal information (Degirmenci 2020). Respondents are least concerned about “AI machines becoming more intelligent than humans” (ranked 10, agree), and about “AI machines turning against and trying to destroy humanity” (ranked 11, agree). This could be due to personal experiences, which are already gained by early AI technologies such as smart home appliances; more advanced AI technology that could become a threat to humanity might be too distant of a scenario.

We collected qualitative responses from our participants, and one participant indicated: “AI is already here but at very early stages”. Other participants emphasised the security and privacy risks: “Extremely concerned about personal data security”, and “high priority is security and privacy concerns”. One of our participants indicated the importance of access to personal information through AI technologies to increase the effectiveness; therefore, we could identify that privacy issues with the diffusion of AI technologies is an important topic to help increasing the effectiveness of AI: “It would seem that the effectiveness of AI will depend on the public’s willingness to hand over their dataset”.

Although the respondents are concerned about risks caused by AI technology, most respondents nevertheless trust AI in their lifestyle. Our respondents mainly trust AI in the workplace (ranked 1, agree) and at home (ranked 2, agree). We found least trust regarding participants’ trust towards companies developing and commercialising AI (ranked 5, agree), and towards government agencies using AI (ranked 6, agree). One explanation we found in our qualitative data was that stronger regulations might help to increase the public perception of trust in AI. One participant argued that “it is a subject that needs regulation before implementing instead of chasing technology after it is introduced”. Another participant also emphasised regulatory aspects: “In some situations AI can be very beneficial, but I think there needs to be some quite strong regulation with it to ensure that society does not become too dependent upon it or that it replaces human relations and employment”.

In terms of AI benefits, factor analysis revealed two factors: (a) AI benefits for urban services and (b) AI benefits for disaster management. Regarding AI benefits for urban services, most of our participants found it beneficial that “AI can help local governments monitor and respond to problems associated with urban infrastructure” (ranked 1, agree), and “AI can help with efficient delivery of urban services” as well as “AI can help local governments monitor and respond to the environmental and climate crises” (both ranked 2 at the same percentage level, agree). Least beneficial were perceived to be that “AI can be used to reduce public sector costs with savings used to reduce rates and other taxes”, and “AI can help improve objectivity in the delivery of urban services” (both ranked 7, agree).

In terms of AI benefits for disaster management, most of our participants agreed that “AI can help in emergency services in disaster-related information gathering” (ranked 1, agree), and “AI can help in predicting disasters, and providing early warnings”. The level of agreement was least for the statement that “AI can help in social media analytics to obtain public perception on disasters” (ranked 9, agree), and “AI can be used in gaming applications to increase community disaster awareness” (ranked 10, agree). These findings are in line with the findings of a recent study on AI and disaster management (Kankanamge et al. 2021).

One participant explained the role of the public interest: “If public interest and ideas are taken to work in the best interest for the community that would work best”, and another participant emphasised the importance of control for AI to truly unfold benefits: “Introduction of AI must be controlled by responsible elected representatives. All AI activities must be monitored by an independent agency appointed by the people”. In terms of AI benefits for environmental improvements, one of our participants indicated the following: “I think having AI focus on the environment would be very smart. But to make sure they are pro-environment as opposed to pro-economy/development at any cost”. This statement reveals an important aspect of conflicting priorities regarding AI benefits, but for whom or for which purpose. While AI technology can provide pro-economic advantages, these might conflict with pro-environmental priorities, which is one promising field of emerging and future research (Truby 2020; Vinuesa et al. 2020).

To compare perception differences between Sydney, Melbourne, and Brisbane, we split the data and conducted further descriptive and factor analyses for the three cities (Appendix C, D, E). Participants from all three cities agree mostly that “AI machines being used to monitor your activity without your permission/knowledge” poses the highest risk (ranked 1, agree). While participants from Sydney and Brisbane agree that “AI machines being used to invade your privacy” is another high risk (ranked 2, agree), Melbourne participants agree that “AI machines being hacked and stealing/losing large amounts of your private data” comes second as a risk (ranked 2, agree). In both cases, we can see that participants from all three cities are mostly concerned with risks related to privacy issues.

In terms of AI trust, there is a notable difference between Sydney and Brisbane on the one hand, and Melbourne on the other hand. While Sydney and Brisbane participants trust mostly AI in the workplace (ranked 1, agree), Melbourne participants trust mostly companies developing and commercialising AI (ranked 1, agree). This is surprising because companies developing and commercialising AI are trusted the least by participants from Sydney (ranked 6, agree), and they are also less trusted by participants from Brisbane (ranked 4, agree).

Regarding AI benefits for urban services, participants from all three cities agree that it is most important that “AI can help local governments monitor and respond to problems associated with urban infrastructure” (ranked 1, agree). However, Brisbane participants perceive it equally important that “AI can be used to monitor urban areas and ensure safety and security of all residents” (ranked 1, agree). This is less important for Sydney participants (ranked 5, agree) and for Melbourne participants as well (ranked 4, agree).

Finally, a comparison revealed further insights for AI benefits for disaster management. While emergency services in disaster-related information gathering is the most important AI benefit for disaster management for participants from Melbourne and Brisbane (ranked 1, agree), Sydney participants perceive that it is more important that “AI can help in disaster response and emergency services in rescue operations” and that “AI can help in determining disaster damages and risky constructions and locations” (both ranked 1, agree). For Melbourne participants, it comes second that “AI can help in increasing effectiveness and efficiency of planning and preparedness for disasters” (ranked 2, agree), and for Brisbane participants, the next most important AI benefit for disaster management is that “AI can help in predicting disasters, and providing early warnings” (ranked 2, agree).

As a next step, we converted the scales from the factor analysis to composite variables. In terms of the factors that drive the public perceptions of AI, we conducted multiple regression analyses for AI risks, AI trust, AI benefits for urban services, and AI benefits for disaster management as dependent variables, and gender, age, education, employment, income, AI knowledge, and AI experience as potential drivers for the independent variables (see Table 2).

**Table 2:** Path coefficients and significance values of drivers impacting AI perceptions (combined dataset for Sydney, Melbourne, and Brisbane)

Drivers	AI perceptions			
	AI risks (*R*^2^ = 0.038)	AI trust (*R*^2^ = 0.106)	AI benefits for urban services (*R*^2^ = 0.086)	AI benefits for disaster management (*R*^2^ = 0.022)
Gender	− 0.105*	0.084*	− 0.034^n.s^	− 0.028^n.s^
Age	0.115*	− 0.228***	0.112*	− 0.052^n.s^
Education	− 0.079^n.s^	− 0.023^n.s^	0.050^n.s^	− 0.011^n.s^
Employment	− 0.002^n.s^	− 0.008^n.s^	0.039^n.s^	− 0.031^n.s^
Income	− 0.054^n.s^	0.084^n.s^	− 0.037^n.s^	0.060^n.s^
AI knowledge	0.129**	0.041^n.s^	0.088^n.s^	− 0.035^n.s^
AI experience	− 0.068^n.s^	0.115*	0.254***	− 0.136**

***p < 0.001; **p < 0.01; *p < 0.05

^n.s^Not significant

The analysis has found that for AI risks, AI knowledge is the most significant driver (*β* = 0.129, *p* < 0.01), while gender (*β* = − 0.105, *p* < 0.05) and age (*β* = 0.115, *p* < 0.05) are also significant. From this, we can conclude that people with more knowledge about AI will perceive AI to be riskier, while female and older individuals will be more prone to AI risks. Regarding AI trust, age is the most significant driver (*β* = − 0.228, *p* < 0.001), while gender (*β* = 0.084, *p* < 0.05) and AI experience (*β* = 0.115, *p* < 0.05) are also significant. We can conclude that with increasing age, AI trust will decrease, while male and more experienced individuals will put more trust in AI.

AI experience is also an important factor for AI benefits for urban services (*β* = 0.254, *p* < 0.001) and also for AI benefits for disaster management (*β* = − 0.136, *p* < 0.01), being the most significant driver for both dependent variables. While there are no other significant drivers for AI benefits for disaster management, age is also significant for AI benefits for urban services (*β* = 0.112, *p* < 0.05). It is surprising that AI experience has a negative effect on AI benefits for disaster management, but this might be since disaster management is a specific context of AI benefits, and the AI experience does not imply that individuals with higher AI experience have experience with AI for disaster management. In terms of AI benefits for urban services, we can conclude that with increasing AI experience, the perception of AI benefits for urban services will increase as well. Further, our results show that older individuals will perceive higher AI benefits for urban services.

Again, we split the data and conducted further multiple regression analyses for Sydney, Melbourne, and Brisbane separately (Appendix F, G, H). Most notable differences include that while gender (*β* = − 0.259, *p* < 0.001) and AI knowledge (*β* = 0.191, *p* < 0.05) are significant drivers for AI risks for Brisbane participants, these factors have no significant impact for Sydney and Melbourne participants. We can observe further differences in perceptions of AI trust. While gender has the strongest impact on AI trust for Sydney participants (*β* = 0.204, *p* < 0.01), age is the most important factor for Melbourne (*β* = − 0.219, *p* < 0.05) and Brisbane participants (*β* = − 0.236, *p* < 0.01). Further, income plays a significant role for AI trust for Melbourne participants (*β* = 0.205, *p* < 0.05). In terms of AI benefits for urban services, age (*β* = 0.281, *p* < 0.01) and AI knowledge (*β* = 0.271, *p* < 0.01) are the most significant drivers for Sydney participants, while for Melbourne participants they are AI experience (*β* = 0.339, *p* < 0.001) and age (*β* = 0.212, *p* < 0.05), and for Brisbane participants AI experience (*β* = 0.226, *p* < 0.01), employment (*β* = 0.227, *p* < 0.05), and age (*β* = − 0.088, *p* < 0.05). Finally, the results show differences regarding AI benefits for disaster management. While AI experience is a significant driver for Melbourne participants (*β* = − 0.184, *p* < 0.05), no significant drivers could be identified for Sydney and Brisbane participants.

In sum, while public perceptions vary depending on the local context, the analysis disclosed the key drivers behind the public perception as gender, age, AI knowledge, and experience.

## Discussion and conclusion

The literature and media regularly report on the exponential developments of AI capabilities and brings our attention to the disruption it is causing and will continue to cause on the way businesses run, government, local services delivered, society function, and the like (Kile 2013; Makridakis 2017). Given the increasing AI disruption in many fronts of public life, government authorities need to pay an increasing attention to public opinions and sentiments towards AI (Cath et al. 2018; Gao et al. 2020; Lee et al. 2020). Furthermore, as stated by Zhai et al. (2020, 140), “public perceptions and concerns about AI are important because the success of any emergent technology depends in large part on public acceptance”.

Our study findings in the case of Australian cities contribute to the current limited knowledge pool on public perceptions on AI and shed light on the main drivers behind the public perceptions. The empirical analysis findings of the Australian cases revealed following insights.

First, the public is concerned of AI monitoring their activity without their permission and invading their privacy. Several studies have already reported the privacy issue as one of the main hurdles in technology adoption, including AI (Mazurek and Małagocka 2019; Radhakrishnan and Chattopadhyay 2020). While these concerns are valid, particularly when AI practices in some authoritarian regimes are concerned (Stark 2021), they also provide an opportunity to place pressure on public authorities and private sector for the development of more ethical and responsible AI frameworks and applications (Constantinescu et al. 2021; Yigitcanlar et al. 2021a). This finding is in line with other studies that highlighted the public anxiety about AI (Vu & Lim 2021).

Moreover, the increasing concerns around the data privacy issues, particularly during the last few years, are attempted to be addressed by the Australian Government through legislative measures. For example, the Privacy Legislation Amendment (enhancing online privacy and other measures) Bill 2021 reinforced the Privacy Act 1988 (https://www.ag.gov.au/rights-and-protections/privacy). Additionally, Australia’s AI Ethics Framework aimed to ensure AI is safe, secure, and reliable (https://www.industry.gov.au/data-and-publications/australias-artificial-intelligence-ethics-framework). Despite these and other legal frameworks (e.g. Australia’s AI Roadmap, Australia’s AI Action Plan, Data Governance Framework 2021), changes in public perceptions take time and require trust to be built. Hence, as much as legislative attempts, effective practice of the policy is required.

Second, the public is less concerned of AI becoming more intelligent than humans and turning against and destroy humanity. Despite catastrophic AI futures imagined for the humankind by science fiction literature and movies, and some entrepreneurs, e.g. Elon Musk, and scientists, e.g. Stephen Hawking, most of the society seems to develop a more optimistic view of “humans and AI might evolve together” just fine (Miller 2019).

Third, the public trusts AI in their lifestyle, mainly in their workplace and at home. Despite some privacy concerns raised, the convenience and efficiency AI offers make it a household technology for business and personal use. Studies on smart homes provided further insights on the underlining motivational factors for trust in and adoption of digital technologies, including AI. For example, a study by Li et al. (2021) identified the main motivations to adopt smart home technologies—including AI and IoT—as efficiency management, better services, cost savings and benefits, and enhanced quality of life.

Fourth, the public shows less trust towards companies developing and commercialising AI, and government agencies using AI. Lack of trust to AI companies may be linked to the social media company scandals, e.g. Facebook’s intervention in Brexit, US and Brazil presidential elections, and a genocide incited on Facebook in Myanmar (Isaak et al. 2018). Lack of trust to government AI applications may be due to a lack of fairness in using AI for public governance, the lack of transparency, unclear responsibility, and accountability and so on (Cui and Wu 2021). Recent failures due to AI use in government may impose negative impacts on governments and society (Zuiderwijk et al. 2021).

Fifth, the public sees value in what AI currently offers and potentially could bring in urban services and disaster management. Recently conducted studies, by Nili et al. (2022), Sanchez et al. (2022) and Yigitcanlar et al. (2022b), revealed insights into public sector experiences about deploying AI and offer a wide spectrum of AI adoption prospects for local governments as creating efficiencies, tackling complexity, managing repetitive tasks, processes, and decisions, automating routine decisions, minimising errors, and improving productivity that particularly benefit customer services, cybersecurity, policy and decision-making, environmental and development control, service and infrastructure management, urban planning, and performance review.

In terms of disaster management, AI is applied in crisis event detection, understanding public reaction, increasing disaster awareness, assess vulnerabilities, serious games/gamified applications, disaster-related data mining and knowledge management, big data analytics from the web or social web, situation recognition, understanding public reaction, eyewitness identification, crisis communication, launch disaster relief activities, i.e. drone-based disaster relief, human/life sign detecting drones, mental health chatbots, crowd evacuation, damage recognition, detecting socioeconomic recovery, understanding public reactions, damage assessments, and human loss estimation (Kankanamge et al. 2021). It seems to be the use of AI for greater good areas such as disaster management, is perceived less critical/negative than the use of AI for profit areas such as product marketing and sales (De Bruyn et al. 2020; Guha et al. 2021).

Sixth, the literature highlights the role of individual factors in AI perceptions. For instance, according to Vu & Lim (2021), “individual factors were strong in shaping public attitude towards AI” (p. 9). Our study identified these individual factors and disclosed that the main drivers behind the public perception of AI include gender, age, AI knowledge and AI experience differences of individuals. To elaborate this further, people with more AI knowledge perceive AI to be riskier, female, and older individuals are more prone to AI risks, with increasing age, AI trust decreases, and male and more experienced individuals put more trust in AI. These findings are in line with other studies that investigated AI and society in the context of Australia. For instance, according to a study by Selwyn and Gallo-Cordoba (2021, p.9), “males were twice as likely to describe themselves as ‘knowing a lot’ about AI rather than ‘never heard of it’ than females… Respondents over 35 years were almost twice as less likely to describe themselves as ‘knowing a lot’ about AI rather than ‘never heard of it’ than those in the 18–24 years age range”. In other words, in general younger males have a better understanding on and experience with AI, hence, they are more aware of the AI risks, but at the same time less vulnerable to AI threats due to the gained consciousness due to knowledge and experience.

Next, depending on the local context, even in the same country, public perceptions on AI may vary. Our study reported some variations in public perceptions among the largest three Australian cities. The variances could be even bigger in the international context. On that very point, Kelley et al. (2021, p.630) underlined that while “Australian public opinions regarding AI might be similar to those in US and Canada (i.e., both countries with similar Anglophone colonial cultures) … they are less congruent with contrasting cultures such as South Korea, Nigeria and India”. Similarly, in countries where AI is used by the state more authoritatively, such as China’s public surveillance with facial recognition practice, the public is more sceptical to AI (Cui and Wu 2021; Kostka et al. 2021).

While, in recent years, AI attracted massive interest of businesses, governments, and societies across the globe, there is still little known on the drivers behind the public perception of this technology (Lozano et al. 2021). This study shed some light on this understudied area in the case study context of Australian cities. The insights generated from this study inform authorities in developing policies to eradicate minimise the public concerns and maximise the public awareness and education on AI.

Last, when interpreting the findings of the study the following limitations should be noted: (a) while the sample size is adequate for the survey, having larger participant numbers might have surfaced additional perspectives; (b) the study focused on three major cities from Australia, while the statistically representation requirements are met, expanding the study to all geographies of the country might have provided extended insights; (c) there are some representation differences between the study participant characteristics and the actual resident characteristics of the three case cities, this might have some impact on the results; (d) the study findings are only quantitatively assessed, the open-ended questions’ answers are not factored in this paper, as these data will be analysed thematically and will be reported in another paper. However, the authors have read and checked all qualitative responses to make sure they are not contradictory to the findings reported in this paper; and (e) there might be unconscious bias in interpreting the study findings.

Finally, this study has only scratched the surface of gaining understanding on what the public thinks of AI and how these opinions were formed. Our future research, further building on the study at hand, will conduct empirical studies to better understand the levels of societal AI literacy—that is “a set of competencies that enables individuals to critically evaluate AI technologies; communicate and collaborate effectively with AI; and use AI as a tool online, at home, and in the workplace” (Long and Magerko 2020, p. 2)—and recommend policy directions to enhance AI literacy in Australia and overseas.

## Data Availability

Not applicable.

## Appendix A: Survey questionnaire

**Part A** **:** **Your background**

What is your gender?

What is your age?

What is your highest education qualification obtained?

What is your current employment status?

Which industry are you employed in or have a business in?

What is your occupation/job title?

What is your gross weekly income?

What is your residential postcode?

**Part B** **:** **Your prior knowledge on AI**

From which source did you mainly learn what AI is?

What is your first thoughts when you think of AI?

Do you feel like you understand the basic concepts of AI?

Which of the following best describes AI’s abilities?

**Part C** **:** **Your prior experience with AI**

Have you ever interacted with an AI technology?

Which of the following technologies have you used or encountered?

How often would you estimate you interact with the previously identified applications?

How long do you think it will be before AI has a noticeable impact on your daily life?

**Part D** **:** **Your perceived benefits of AI**

Which of the following is the most beneficial function of AI technology?

Which of the following is the most promising about the future use of AI?

**Part E** **:** **Your perceived risks of AI**

Which of the following is the biggest disadvantage of AI technology?

Which of the following do you fear the most about the future use of AI?

**Part F** **:** **Your view on the AI future**

Do you think society will become better or worse as a result of the future use of AI?

Do you think the benefits outweigh the risks when looking at the future use of AI?

**Part G** **:** **Your comfort and trust with AI**

How do you feel about AI in general?

Which of the following would you be comfortable with AI operating autonomously?

Would you feel comfortable in a fully autonomous place to live or work?

How does the prospect of an AI future make you feel?

To what degree do the following scenarios concern you?

How much do you trust AI in your lifestyle?

**Part H** **:** **Your view on AI in local government and urban services**

Which of the following urban services are most suited for the future application of AI technology?

When do you believe AI supported urban services will come into your city?

Where do you think AI is needed the most in local government and urban services?

To what degree would AI be useful in the following?

Which of the following are the key challenges for local governments to adopt AI for local service delivery?

**Part I** **:** **Your view on AI in disaster management**

In which areas would AI be useful in disaster (bushfires, flooding, landslides, severe storms) management?

To what degree would AI be useful in these disaster (bushfires, flooding, landslides, severe storms) related issues?

## Appendix B: Demographics

**Table:**

Attribute	Category	Frequency	Survey %	Census %
City	Sydney	201	33.2	–
	Melbourne	201	33.2	–
	Brisbane	203	33.6	–
Age	18–24	66	10.9	
	25–34	117	19.3	
	35–44	126	20.8	
	45–54	115	19.0	
	55–64	81	13.4	
	65–74	45	7.4	
	75–84	31	5.1	
	85 +	24	4.0	
Gender	Female	304	50.2	50.7
	Male	299	49.4	49.3
	Other	2	0.3	–
Education	Year 10 or below	49	8.1	18.8
	Year 11 or equivalent	9	1.5	4.9
	Year 12 or equivalent	88	14.5	15.7
	Certificate	83	13.7	15.8
	Advanced diploma/diploma	85	14.0	8.9
	Bachelor degree	193	31.9	22.0
	Postgraduate degree	98	16.2	–
Employment status	Employed	372	61.5	93.1
	Unemployed	85	14.0	6.9
	Not in labour force	73	12.1	–
	Retired	75	12.4	
Industry	Accommodation, hospitality, and food services	37	6.1	
	Administration and support services	33	5.5	
	Agriculture, forestry, and fishing	2	0.3	
	Arts and recreation	13	2.1	
	Construction	32	5.3	
	Education and training	58	9.6	
	Electricity, gas, water, and waste services	6	1.0	
	Financial and insurance services	26	4.3	
	Healthcare and social assistance	50	8.3	
	Information, media, and telecommunications	33	5.5	
	Manufacturing	27	4.5	
	Mining	1	0.2	
	Mining and natural resources	2	0.3	
	Professional, scientific, and technical services	45	7.4	
	Public administration and safety	23	3.8	
	Rental, hiring, and real estate services	6	1.0	
	Retail trade	55	9.1	
	Transport, postal, and warehousing	23	3.8	
	Wholesale trade	11	1.8	
	Other	90	14.9	
Occupation	Professional	107	17.7	22.2
	Clerical or office worker	90	14.9	13.6
	Manager, executive or director	59	9.8	13.0
	Sales worker	41	6.8	9.4
	Skilled tradesperson	21	3.5	
	Unskilled or labourer	31	5.1	9.5
	Consultant	23	3.8	
	Semi-skilled worker	40	6.6	
	Technology professional	14	2.3	
	Student	40	6.6	
	Manufacturer	11	1.8	
	Agriculture and fisheries employee	2	0.3	
	Business owner	20	3.3	
	Homemaker	14	2.3	
	Other	91	15.0	
Income (weekly gross)	No income	68	11.2	
	$1–$499	95	15.7	
	$500–$999	151	25.0	
	$1,000-$1,999	177	29.3	
	$2,000–$2,999	74	12.2	
	$3,000 or more	40	6.6	

## Appendix C: Results of the descriptive and factor analyses for Sydney

**Table:**

Attributes	Descriptive analysis					Factor analysis			
	DA%	Neutral%	A%	R-DA	R-A	AI risks	AI trust	AI benefits for urban services	AI benefits for disaster management
AI machines making misdiagnosing illness	14.9	27.4	57.7		5	0.843			
AI machines creating mass unemployment	14.4	28.4	57.2		6	0.805			
AI machines making bias decisions	15.9	29.9	54.2		8	0.793			
AI machines replacing your job	19.9	24.4	55.7		7	0.782			
AI machines being unreliable	14.4	31.8	53.7		9	0.777			
AI machines being used for terrorist activity	12.4	28.9	58.7		4	0.768			
AI machines being used to monitor your activity without your permission/knowledge	8.5	20.4	71.1		1	0.754			
AI machines being used to invade your privacy	10.4	22.4	67.2		2	0.745			
AI machines turning against and trying to destroy humanity	29.9	28.4	41.8		11	0.735			
AI machines becoming more intelligent than humans	22.9	27.9	49.3		10	0.697			
AI machines being hacked and stealing/losing large amounts of your private data	11.9	21.4	66.7		3	0.662			
How do you feel about the use of AI in public spaces?	26.9	32.3	40.8		3		0.846		
How do you feel about the corporations using AI?	26.4	34.3	39.3		4		0.812		
How do you feel about the government agencies using AI?	30.3	30.3	39.3		4		0.811		
How do you feel about the companies developing and commercialising AI?	23.4	40.8	35.8		6		0.786		
How do you feel about the use of AI in your workplace?	24.4	30.8	44.8		1		0.777		
How do you feel about the use of AI in your home?	27.9	30.3	41.8		2		0.714		
AI can help with efficient delivery of urban services	7.0	32.3	60.7		4			0.842	
AI can help local governments monitor and respond to problems associated with urban infrastructure	7.0	29.9	63.2		1			0.837	
AI can help improve objectivity in the delivery of urban services	7.0	35.3	57.7		7			0.800	
AI can free up resources so that local governments can spend more time focusing on resident needs and concerns	7.5	30.8	61.7		3			0.795	
AI can be used to monitor urban areas and ensure safety and security of all residents	9.0	31.8	59.2		5			0.773	
AI can be used to reduce public sector costs with savings used to reduce rates and other taxes	11.4	31.8	56.7		8			0.729	
AI can help local governments monitor and respond to the environmental and climate crises	9.5	28.9	61.7		2			0.728	
AI can be used to reduce public sector costs with savings to improve other urban services	9.5	31.8	58.7		6			0.713	
AI can help in determining disaster hotspots and severity	21.4	24.9	53.7		5				0.806
AI can help in emergency services in disaster-related information gathering	23.9	19.9	56.2		3				0.804
AI can help in disaster response and emergency services in rescue operations	19.4	22.9	57.7		1				0.794
AI can help in responding queries of the public regarding to disasters	23.4	26.4	50.2		7				0.770
AI can help in increasing effectiveness and efficiency of planning and preparedness for disasters	21.4	23.4	55.2		4				0.743
AI can help in determining disaster damages and risky constructions and locations	21.4	20.9	57.7		1				0.743
AI can help in identifying fake news about disasters	22.9	26.9	50.2		7				0.717
AI can help in predicting disasters, and providing early warnings	21.4	24.9	53.7		5				0.704
AI can help in social media analytics to obtain public perception on disasters	23.9	28.9	47.3		9				0.672
AI can be used in gaming applications to increase community disaster awareness	28.4	30.3	41.3		10				0.601
Sum of squared loadings (rotated)						6.579	4.667	6.293	5.545
% of variance explained						18.799	13.335	17.981	15.842
Reliability (Standard Cronbach’s alpha)						0.930	0.950	0.939	0.906
Kaiser–Meyer–Olkin (KMO)									0.876
N									201
Extraction method						Principal component analysis			
Rotation method						Varimax with Kaiser Normalisation			

*DA*  disagree, *A*   agree, *R*  rank

## Appendix D: Results of the descriptive and factor analyses for Melbourne

**Table:**

Attributes	Descriptive analysis					Factor analysis			
	DA%	Neutral%	A%	R-DA	R-A	AI risks	AI trust	AI benefits for urban services	AI benefits for disaster management
AI machines being hacked and stealing/losing large amounts of your private data	13.4	25.4	61.2		2	0.843			
AI machines being used to monitor your activity without your permission/knowledge	9.0	29.4	61.7		1	0.824			
AI machines being used to invade your privacy	11.9	27.9	60.2		3	0.808			
AI machines being unreliable	16.9	36.8	46.3		9	0.804			
AI machines creating mass unemployment	18.9	27.9	53.2		5	0.781			
AI machines making bias decisions	12.9	36.8	50.2		7	0.757			
AI machines being used for terrorist activity	13.4	32.3	54.2		4	0.751			
AI machines replacing your job	23.4	28.4	48.3		8	0.746			
AI machines turning against and trying to destroy humanity	34.3	29.9	35.8		11	0.741			
AI machines making misdiagnosing illness	12.4	35.3	52.2		6	0.705			
AI machines becoming more intelligent than humans	27.4	29.4	43.3		10	0.635			
How do you feel about the government agencies using AI?	29.9	34.3	35.8		6		0.853		
How do you feel about the corporations using AI?	23.9	39.3	36.8		5		0.844		
How do you feel about the companies developing and commercialising AI?	23.4	37.8	38.8		1		0.829		
How do you feel about the use of AI in your workplace?	24.4	37.3	38.3		2		0.814		
How do you feel about the use of AI in your home?	27.4	35.3	37.3		4		0.805		
How do you feel about the use of AI in public spaces?	22.4	39.8	37.8		3		0.747		
AI can be used to reduce public sector costs with savings to improve other urban services	11.4	37.3	51.2		6			0.882	
AI can help local governments monitor and respond to problems associated with urban infrastructure	8.0	31.8	60.2		1			0.873	
AI can help local governments monitor and respond to the environmental and climate crises	10.0	31.8	58.2		2			0.858	
AI can be used to reduce public sector costs with savings used to reduce rates and other taxes	12.4	35.8	51.7		5			0.857	
AI can free up resources so that local governments can spend more time focusing on resident needs and concerns	11.9	36.8	51.2		6			0.854	
AI can help with efficient delivery of urban services	8.5	34.3	57.2		3			0.842	
AI can be used to monitor urban areas and ensure safety and security of all residents	14.4	30.3	55.2		4			0.790	
AI can help improve objectivity in the delivery of urban services	11.9	39.8	48.3		8			0.750	
AI can help in increasing effectiveness and efficiency of planning and preparedness for disasters	26.9	25.9	47.3		2				0.873
AI can help in disaster response and emergency services in rescue operations	28.9	24.4	46.8		3				0.842
AI can help in emergency services in disaster-related information gathering	25.9	22.9	51.2		1				0.830
AI can help in responding queries of the public regarding to disasters	26.4	28.4	45.3		5				0.830
AI can help in social media analytics to obtain public perception on disasters	25.4	33.3	41.3		9				0.825
AI can help in predicting disasters, and providing early warnings	29.4	26.4	44.3		7				0.820
AI can help in determining disaster hotspots and severity	28.9	25.9	45.3		5				0.786
AI can help in determining disaster damages and risky constructions and locations	25.9	28.4	45.8		4				0.778
AI can help in identifying fake news about disasters	26.9	30.8	42.3		8				0.767
AI can be used in gaming applications to increase community disaster awareness	30.8	29.4	39.8		10				0.643
Sum of squared loadings (rotated)						6.639	4.722	6.488	6.481
% of variance explained						18.967	13.491	18.537	18.518
Reliability (Standard Cronbach’s alpha)						0.928	0.951	0.957	0.938
Kaiser–Meyer–Olkin (KMO)									0.878
N									201
Extraction method						Principal component analysis			
Rotation method						Varimax with Kaiser Normalisation			

*DA* disagree, *A* agree, *R* = rank

## Appendix E: Results of the descriptive and factor analyses for Brisbane

**Table:**

Attributes	Descriptive analysis					Factor analysis			
	DA%	Neutral%	A%	R-DA	R-A	AI risks	AI trust	AI benefits for urban services	AI benefits for disaster management
AI machines being used to monitor your activity without your permission/knowledge	9.4	18.2	72.4		1	0.828			
AI machines being used to invade your privacy	12.3	16.7	70.9		2	0.825			
AI machines making misdiagnosing illness	13.3	20.2	66.5		4	0.821			
AI machines being used for terrorist activity	16.3	17.7	66.0		5	0.820			
AI machines being hacked and stealing/losing large amounts of your private data	10.3	20.2	69.5		3	0.779			
AI machines being unreliable	20.2	23.6	56.2		8	0.772			
AI machines making bias decisions	12.3	21.7	66.0		5	0.757			
AI machines creating mass unemployment	19.2	19.7	61.1		7	0.684			
AI machines turning against and trying to destroy humanity	34.5	24.1	41.4		11	0.646			
AI machines replacing your job	22.2	22.7	55.2		9	0.603			
AI machines becoming more intelligent than humans	28.6	27.1	44.3		10	0.488			
How do you feel about the companies developing and commercialising AI?	27.6	38.9	33.5		4		0.804		
How do you feel about the use of AI in your home?	31.5	32.5	36.0		2		0.797		
How do you feel about the use of AI in your workplace?	27.6	35.5	36.9		1		0.781		
How do you feel about the use of AI in public spaces?	29.6	34.5	36.0		2		0.778		
How do you feel about the corporations using AI?	31.5	35.0	33.5		4		0.762		
How do you feel about the government agencies using AI?	34.5	33.0	32.5		6		0.739		
AI can help with efficient delivery of urban services	14.3	29.1	56.7		3			0.874	
AI can help local governments monitor and respond to the environmental and climate crises	13.8	31.5	54.7		4			0.870	
AI can free up resources so that local governments can spend more time focusing on resident needs and concerns	16.3	36.5	47.3		8			0.870	
AI can be used to reduce public sector costs with savings used to reduce rates and other taxes	15.8	34.5	49.8		7			0.856	
AI can be used to reduce public sector costs with savings to improve other urban services	16.3	32.5	51.2		6			0.853	
AI can help local governments monitor and respond to problems associated with urban infrastructure	13.8	28.1	58.1		1			0.838	
AI can help improve objectivity in the delivery of urban services	14.8	33.0	52.2		5			0.821	
AI can be used to monitor urban areas and ensure safety and security of all residents	13.8	28.1	58.1		1			0.727	
AI can help in increasing effectiveness and efficiency of planning and preparedness for disasters	28.6	23.6	47.8		4				0.809
AI can help in predicting disasters, and providing early warnings	27.1	21.7	51.2		2				0.803
AI can help in disaster response and emergency services in rescue operations	30.0	25.6	44.3		6				0.800
AI can help in emergency services in disaster-related information gathering	25.1	21.2	53.7		1				0.786
AI can help in responding queries of the public regarding to disasters	30.5	27.1	42.4		8				0.779
AI can help in determining disaster hotspots and severity	29.6	21.7	48.8		3				0.779
AI can help in social media analytics to obtain public perception on disasters	27.1	32.5	40.4		9				0.773
AI can help in determining disaster damages and risky constructions and locations	29.1	24.6	46.3		5				0.771
AI can help in identifying fake news about disasters	28.6	28.6	42.9		7				0.695
AI can be used in gaming applications to increase community disaster awareness	30.5	34.0	35.5		10				0.589
Sum of squared loadings (rotated)						6.182	4.280	6.787	5.908
% of variance explained						17.662	12.230	19.390	16.880
Reliability (Standard Cronbach’s alpha)						0.911	0.950	0.957	0.920
Kaiser–Meyer–Olkin (KMO)									0.887
*N*									203
Extraction method						Principal component analysis			
Rotation method						Varimax with Kaiser Normalisation			

*DA* disagree, *A* agree, *R* rank

## Appendix F: Path coefficients and significance values of drivers impacting AI perceptions for Sydney

**Table:**

Drivers	AI perceptions			
	AI risks (*R*^2^ = 0.025)	AI trust (*R*^2^ = 0.214)	AI benefits for urban services (*R*^2^ = 0.132)	AI benefits for disaster management (*R*^2^ = 0.023)
Gender	0.007^n.s^	0.204**	− 0.020^n.s^	− 0.039^n.s^
Age	0.147^n.s^	− 0.167*	0.281**	0.035^n.s^
Education	− 0.078^n.s^	0.036^n.s^	− 0.001^n.s^	− 0.061^n.s^
Employment	− 0.057^n.s^	− 0.197*	− 0.074^n.s^	− 0.095^n.s^
Income	− 0.054^n.s^	− 0.052^n.s^	− 0.063^n.s^	0.012^n.s^
AI knowledge	0.090^n.s^	0.183*	0.271**	0.033^n.s^
AI experience	− 0.047^n.s^	0.012^n.s^	0.136^n.s^	− 0.144^n.s^

****p* < 0.001; ***p* < 0.01; **p* < 0.05

^n.s^Not significant

## Appendix G: Path coefficients and significance values of drivers impacting AI perceptions for Melbourne

**Table:**

Drivers	AI perceptions			
	AI risks (*R*^2^ = 0.052)	AI trust (*R*^2^ = 0.106)	AI benefits for urban services (*R*^2^ = 0.134)	AI benefits for disaster management (*R*^2^ = 0.045)
Gender	− 0.071^n.s^	0.026^n.s^	− 0.050^n.s^	0.060^n.s^
Age	0.043^n.s^	− 0.219*	0.212*	− 0.144^n.s^
Education	− 0.143^n.s^	− 0.049^n.s^	0.063^n.s^	0.055^n.s^
Employment	− 0.014^n.s^	0.047^n.s^	− 0.081^n.s^	0.006^n.s^
Income	− 0.114^n.s^	0.205*	− 0.137^n.s^	0.040^n.s^
AI knowledge	0.116^n.s^	− 0.041^n.s^	0.094^n.s^	− 0.087^n.s^
AI experience	− 0.079^n.s^	0.141^n.s^	0.339***	− 0.184*

****p* < 0.001; ***p* < 0.01; **p* < 0.05

^n.s^Not significant

## Appendix H: Path coefficients and significance values of drivers impacting AI perceptions for Brisbane

**Table:**

Drivers	AI perceptions			
	AI risks (*R*^2^ = 0.096)	AI trust (*R*^2^ = 0.086)	AI benefits for urban services (*R*^2^ = 0.082)	AI benefits for disaster management (*R*^2^ = 0.038)
Gender	− 0.259***	0.038^n.s^	− 0.012^n.s^	− 0.110^n.s^
Age	0.134^n.s^	− 0.236**	− 0.088*	− 0.064^n.s^
Education	− 0.050^n.s^	− 0.029^n.s^	0.067^n.s^	− 0.091^n.s^
Employment	0.081^n.s^	0.029^n.s^	0.227*	− 0.010^n.s^
Income	0.010^n.s^	0.040^n.s^	0.080^n.s^	0.159^n.s^
AI knowledge	0.191*	0.006^n.s^	− 0.008^n.s^	0.009^n.s^
AI experience	− 0.064^n.s^	0.154*	0.226**	− 0.102^n.s^

****p* < 0.001; ***p* < 0.01; **p* < 0.05

^n.s^Not significant.
